# Supplementary material for: Reprogramming of human cells to pluripotency induces CENP-A chromatin depletion
Source: Open Biol. 2020 Oct 21;10(10):200227. doi: 10.1098/rsob.200227 (PMC7653353; doi:10.1098/rsob.200227)
Supplement: Supplemental Figure 1 [file rsob200227supp1.pdf]

Supplemental Information

Reprogramming of Human Cells to Pluripotency Induces CENP-A Chromatin Depletion

Inês Milagre, Carolina Pereira, Raquel A. Oliveira and Lars E.T. Jansen

- **1 Supplemental figure with corresponding legend**

Figure S1

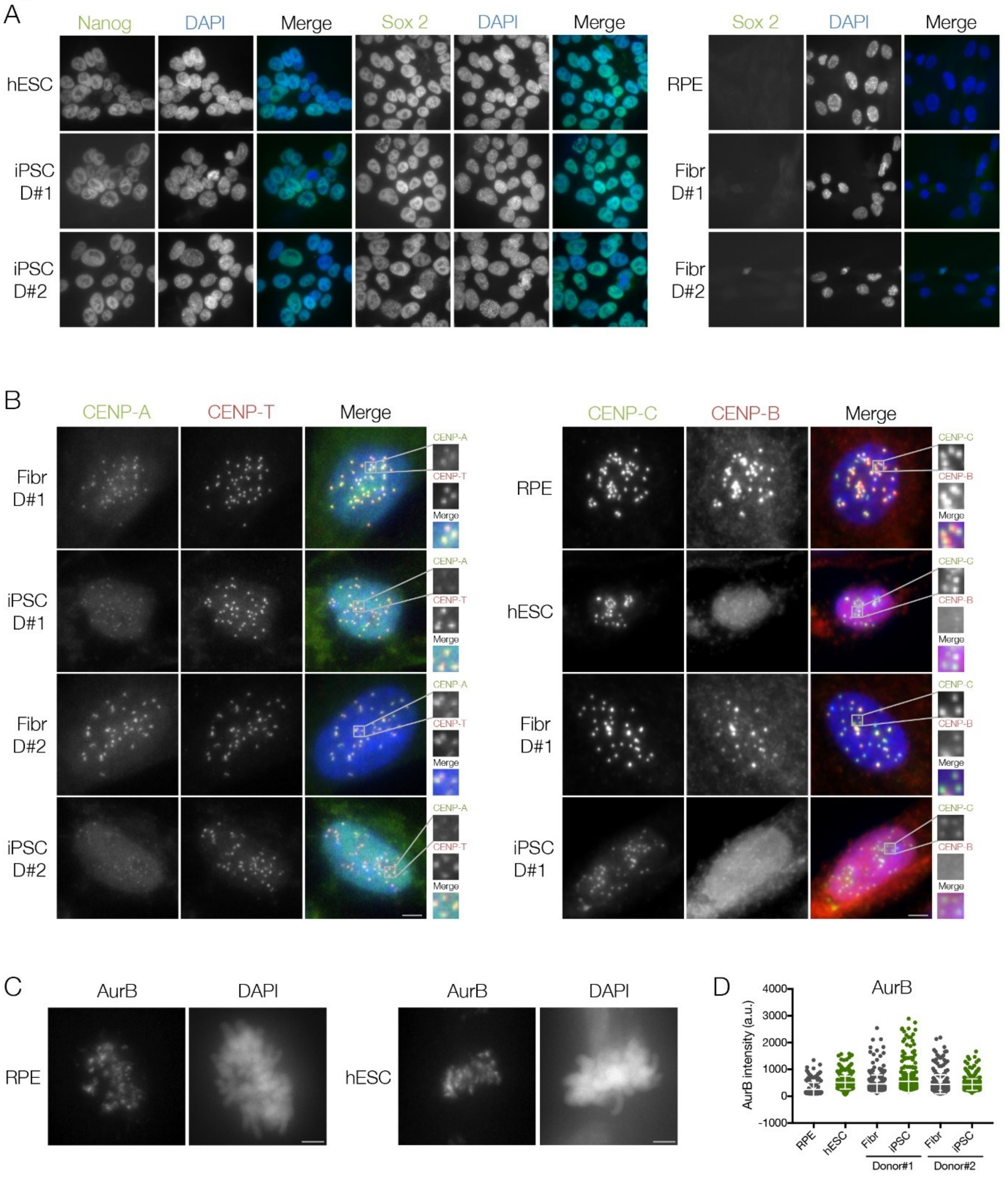

Figure S1. Pluripotent stem cells have a weaker centromere, but normal levels of AurB, when compared to differentiated cells. A), B) Differentiated (RPE and Fibr D#1 and Fibr D#2) and pluripotent stem cells (hESC or iPSC D#1 or iPSC D#2) were fixed and stained for either A) Nanog or Sox2 and counterstained with DAPI. Representative immunofluorescence images are shown, or B) CENP-A, CENP-T, CENP-C or CENP-B and counterstained with DAPI. Representative immunofluorescence images from Fibroblasts and iPSC from Donor #1 and Donor #2 are shown for CENP-A and CENP-T and representative images from RPE and hESC, Fibroblasts and iPSC from Donor #1 are shown for CENP-B and CENP-C. **C)** Differentiated (RPE and Fibr D#1 and Fibr D#2) and pluripotent stem cells (hESC or iPSC D#1 or iPSC D#2) were fixed and stained for Aurora B (AurB) and counterstained with DAPI. **D)** Quantification of centromere intensities for AurB. Average centromere intensities were determined using automatic centromere recognition and quantification (CRaQ) for indicated cell types. Horizontal lines represent the mean and whiskers represent standard deviation, for each sample. Scale bar = 2µm.
