# Supplementary material for: Reprogramming of human cells to pluripotency induces CENP-A chromatin depletion
Source: Open Biol. 2020 Oct 21;10(10):200227. doi: 10.1098/rsob.200227 (PMC7653353; doi:10.1098/rsob.200227)

Western Blots

Figure 2A

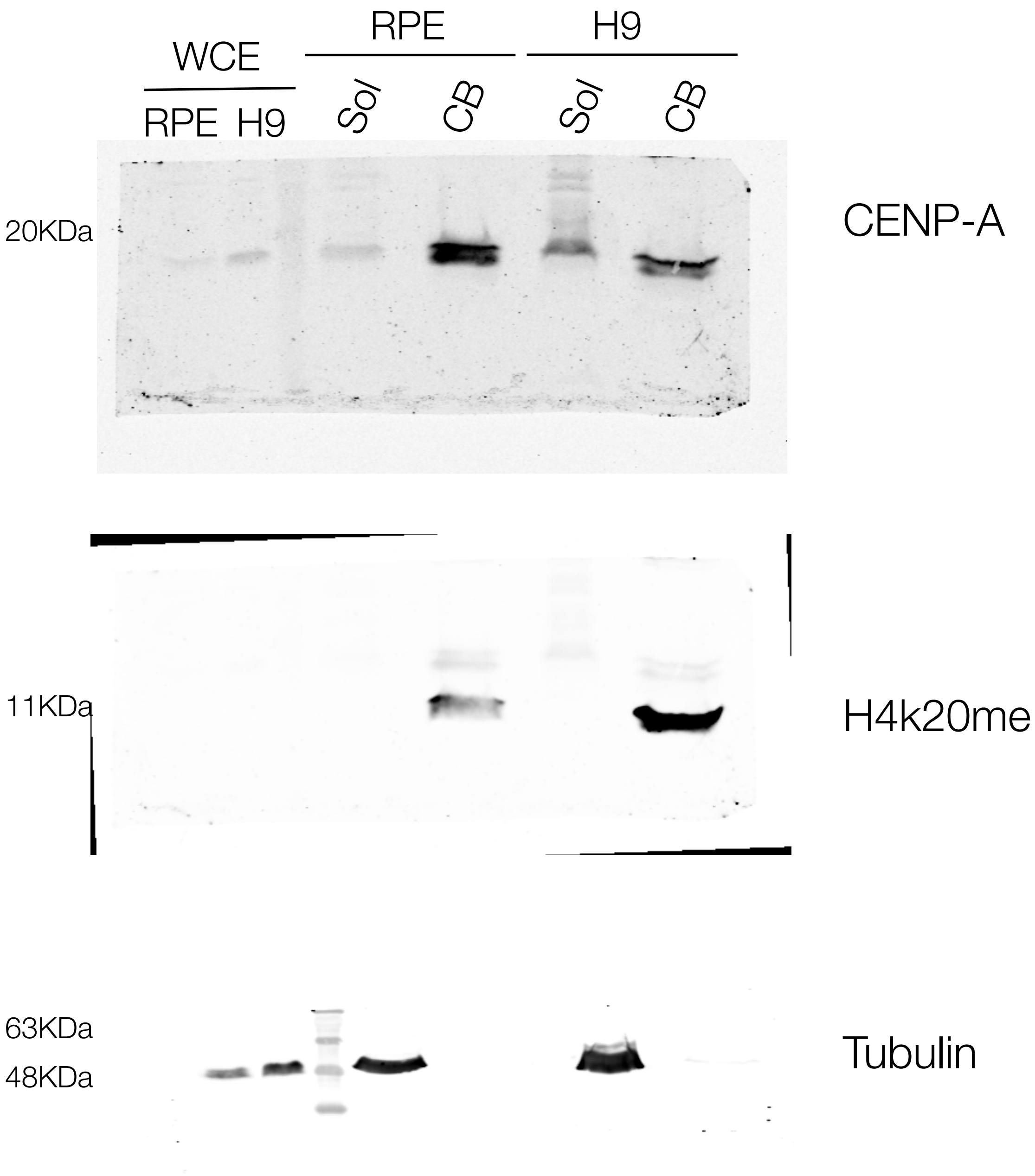

Western Blots

Pair of images consists of two channels of the same blot

Figure 2 C

CENP-A 700 Channel

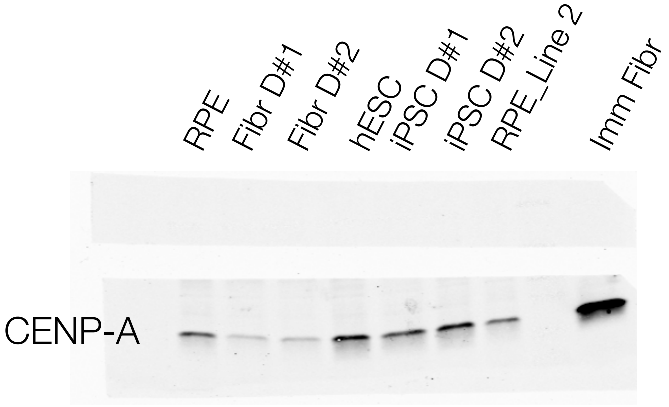

Tubulin 800 Channel

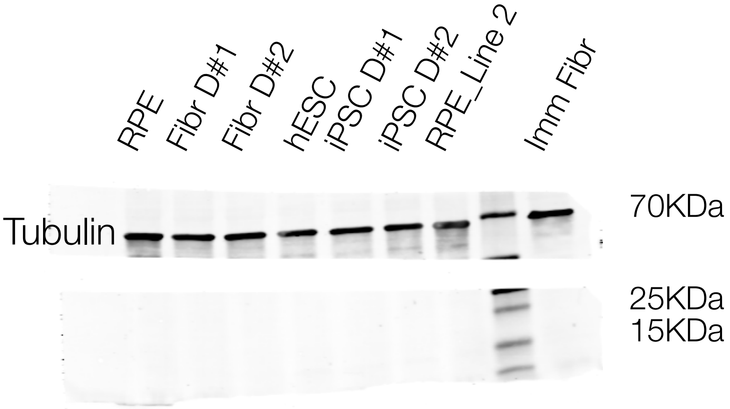

This same gel was used to assess CENP-E (Figure 4)

Pair of images consists of two channels of the same blot

CENP-B 700 Channel

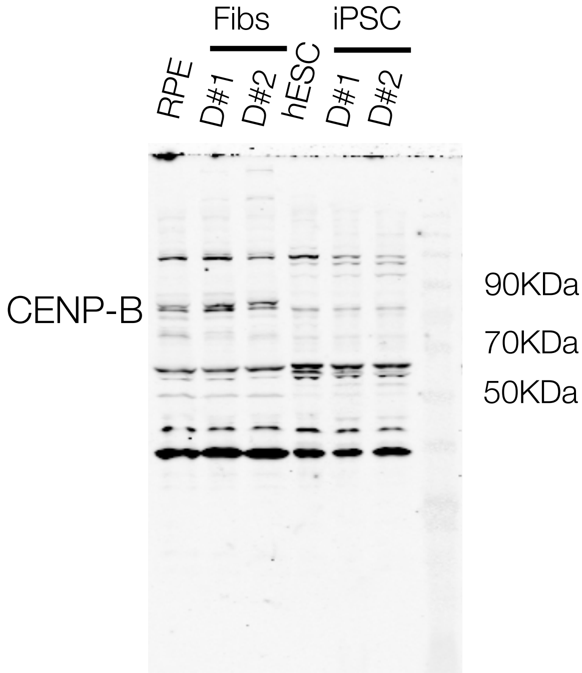

Tubulin 800 Channel

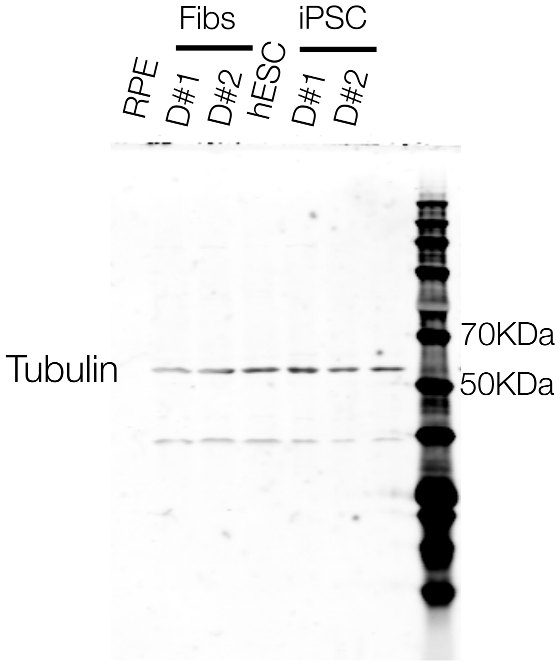

Western Blots

Figure 2 C

Pair of images consists of two channels of the same blot

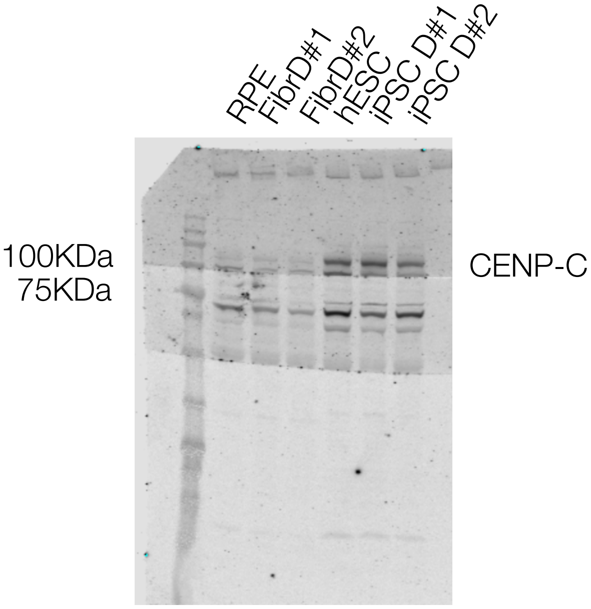

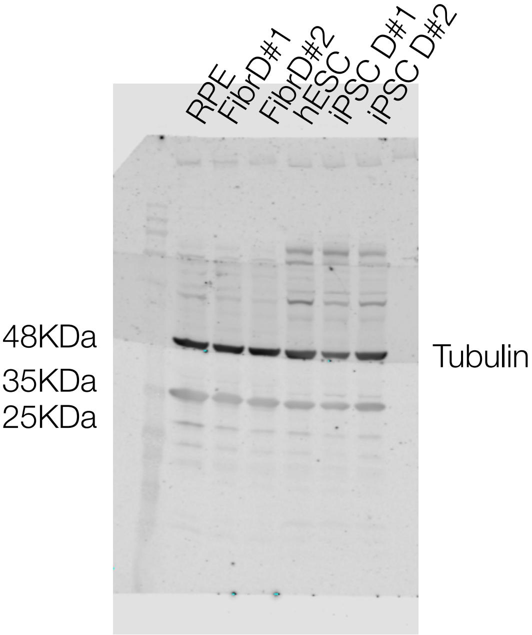

This same gel was used to assess CENP-T

Pair of images consists of two channels of the same blot

CENP-T

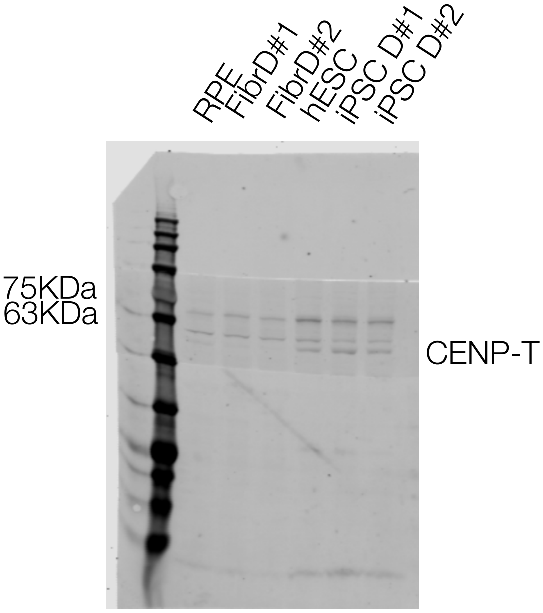

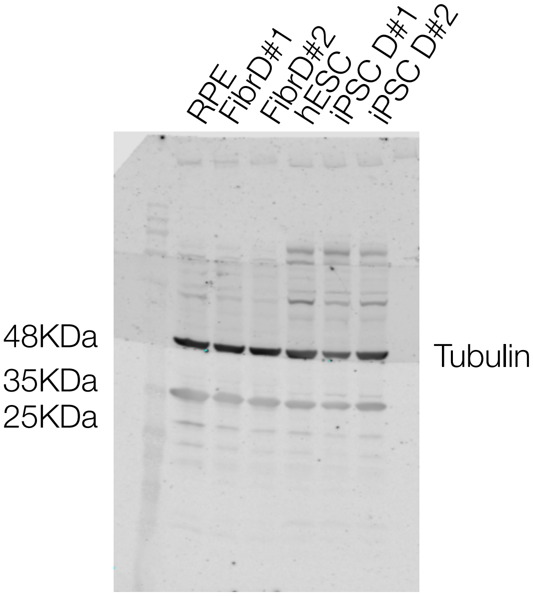

Western Blots

Figure 4

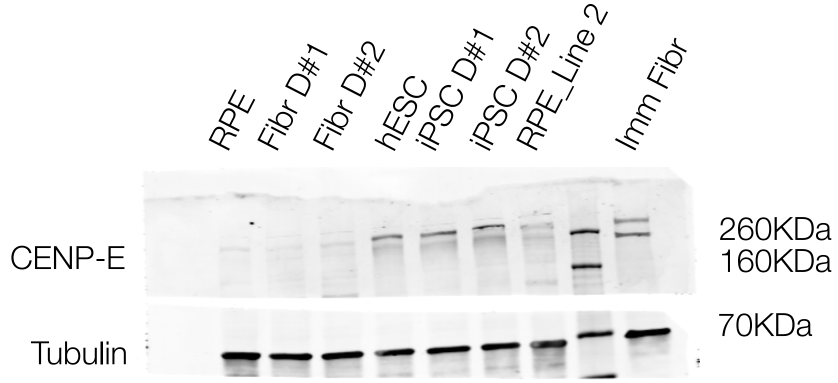

This same gel was used to assess CENP-A (Figure 2)

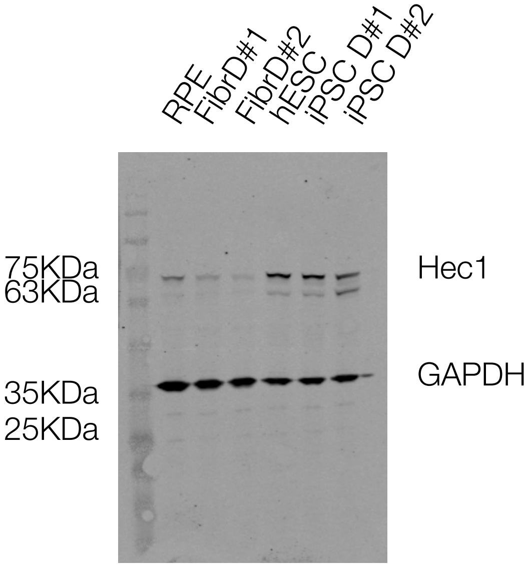

Supplement: Raw annotated Immunoblots [file rsob200227supp2.pdf]
